# Supplementary material for: A Dual-Reporter Platform for Screening Tumor-Targeted Extracellular Vesicles
Source: Pharmaceutics. 2022 Feb 22;14(3):475. doi: 10.3390/pharmaceutics14030475 (PMC8953635; doi:10.3390/pharmaceutics14030475)
Supplement: Supplementary file 1 [file pharmaceutics-14-00475-s001.zip › pharmaceutics-1530192-supplementary.pdf]

pharmaceutics

# Supplementary Materials: A Dual-Reporter Platform for Screening Tumor-Targeted Extracellular Vesicles

Masamitsu Kanada, Lauren Linenfelser, Elyssa Cox and Assaf A. Gilad

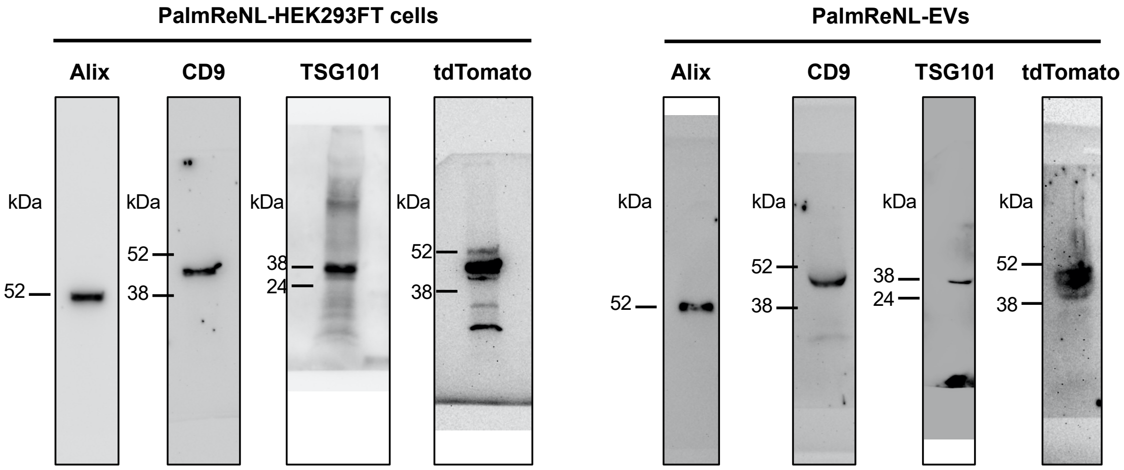


**Figure S1.** Western blot analysis of exosome marker proteins in PalmReNL-HEK293FT cells and -EVs. Original images for Figure 1D.

# DOPE-uPAR (VSNKYFSNIHWGC)

A

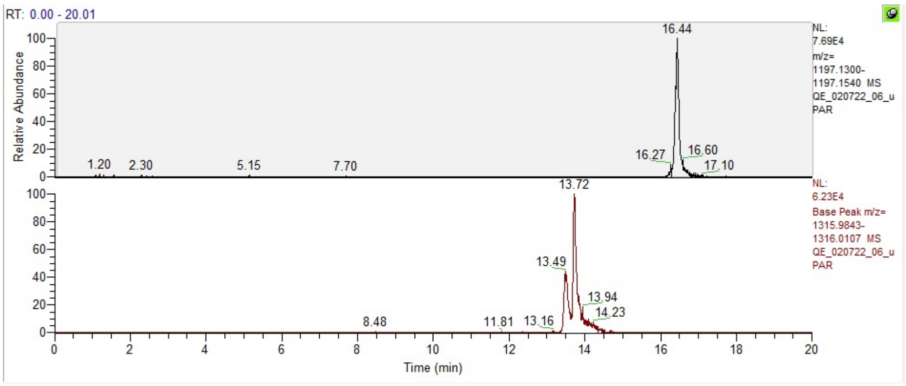


B

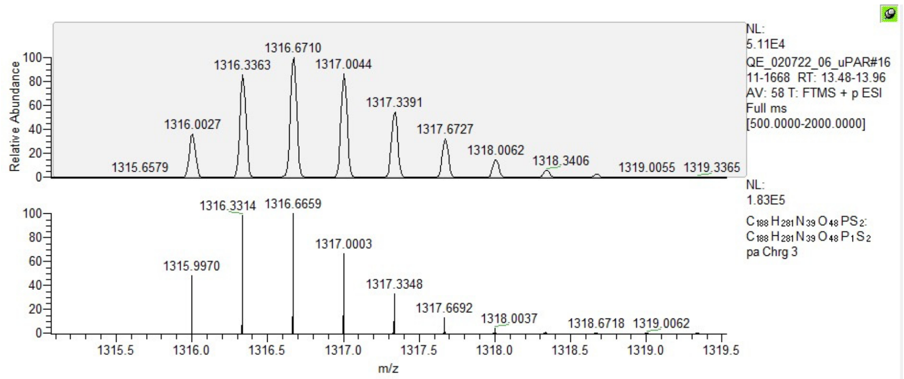


C

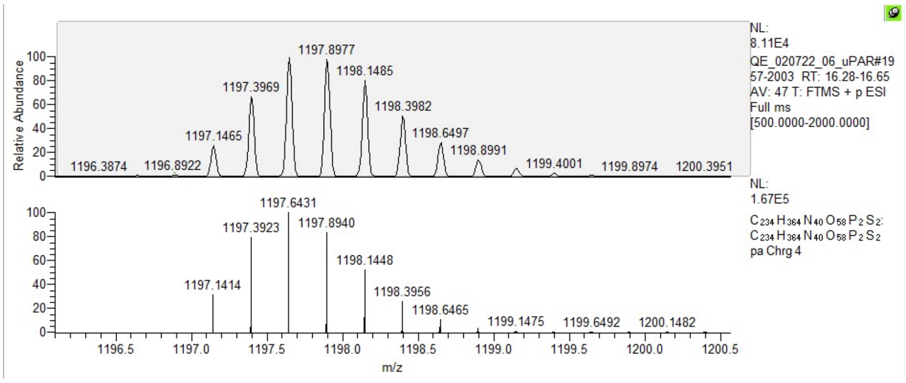

# DOPE-PDL1 (YASYHCWCWRDPGRS)

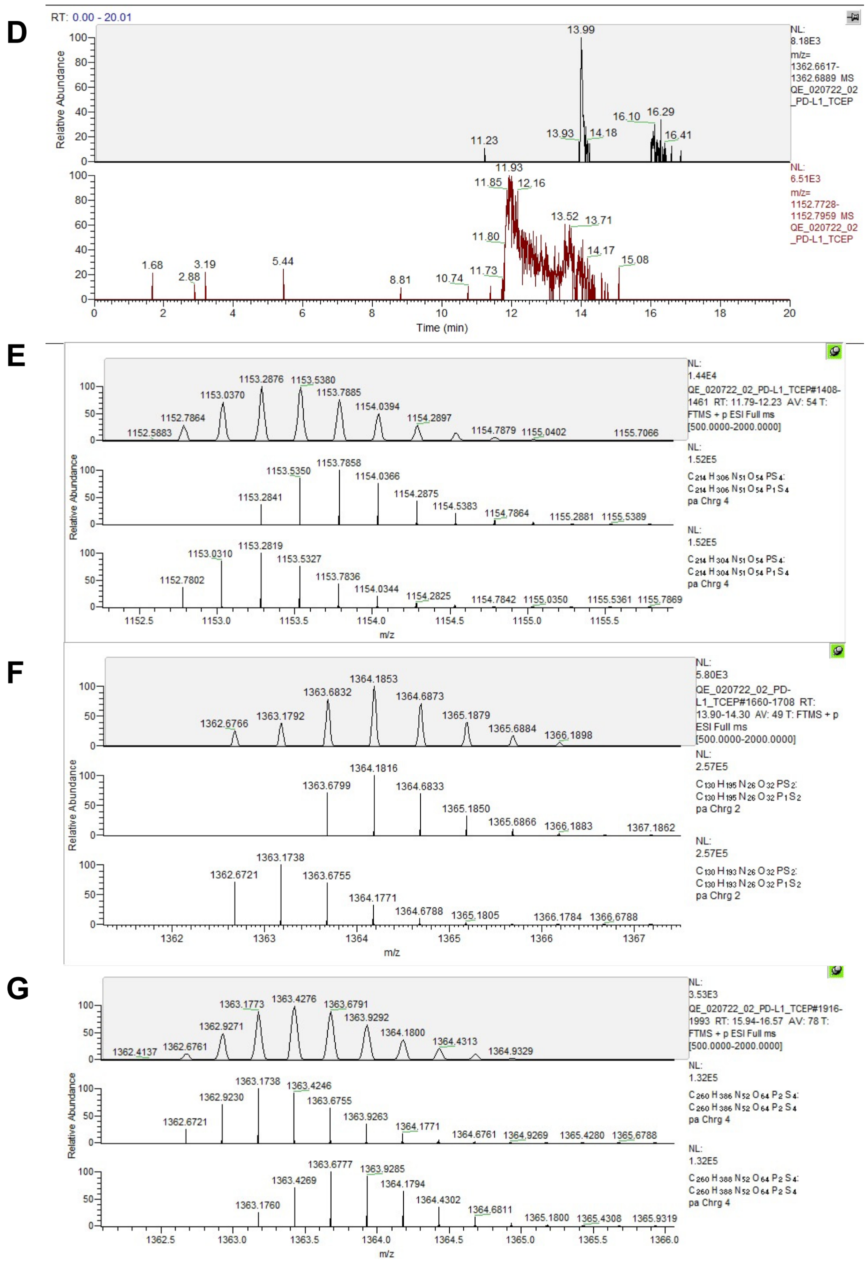

# DOPE-EGFR (KKKGGYHWYGYTPQNVI)

H

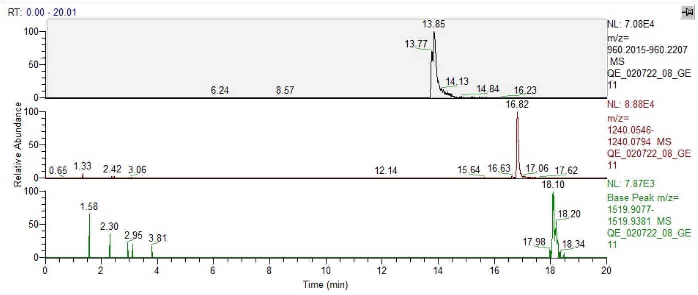


I

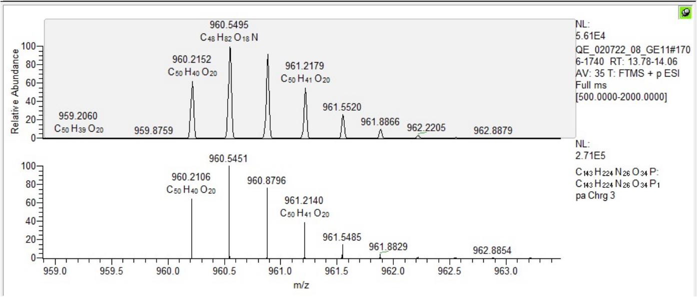


J

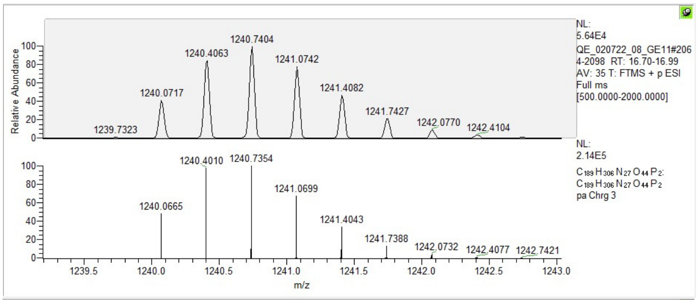


K

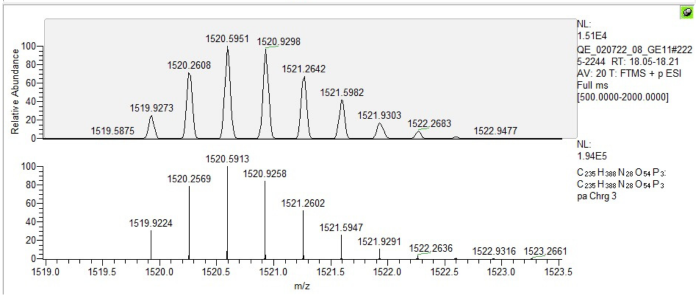


**Figure S2.** Characterization of DOPE-THPs by LC/MS. **A**) Extracted ion chromatograms for m/z 1315.9975 and 1197.142 (+/- 10 ppm). **B**) Mass spectrum at 13.48-13.96 min showing DOPE-uPAR-uPAR-DOPE conjugate with a disulfide linking two DOPE-uPARs. Isotope models are shown below the raw data. **C**) Mass spectrum at 16.28-16.65 min showing DOPE-uPAR-uPAR conjugate with a disulfide linking one DOPE-uPAR and one unmodified uPAR-binding peptide. Isotope models are shown below the raw data. **D**) Extracted ion chromatogram for m/z 1152.7844 and 1362.6753 (+/- 10 ppm). **E**) Mass spectrum at 11.79-12.23 min showing DOPE-PDL1-PD-L1 conjugate with one or two disulfides linking two PD-L1-binding peptides. Isotope models are shown below the raw data. **F**) Mass spectrum at 13.9-14.3 min showing DOPE-PDL1 conjugate with none or one internal disulfide linkage. Isotope models are shown below the raw data. **G**) Mass spectrum at 15.94-16.57 min showing DOPE-PDL1-PDL1-DOPE conjugate with one or two disulfides linking two DOPE-PDL1s. **H**) Extracted ion chromatograms for m/z 960.2111, 1240.067 and 1519.9929 (+/- 10 ppm). **I**) Mass spectrum at 13.78-14.06 min showing one EGFR-binding peptide with one DOPE attached. Isotope models are shown below the raw data. **J**) Mass spectrum at 16.70-16.99 min showing one EGFR-binding peptide with two DOPE molecules attached. Isotope models are shown below the raw data. **K**) Mass spectrum at 18.05-18.21 min showing one EGFR-binding peptide with three DOPE molecules attached. Isotope models are shown below the raw data.

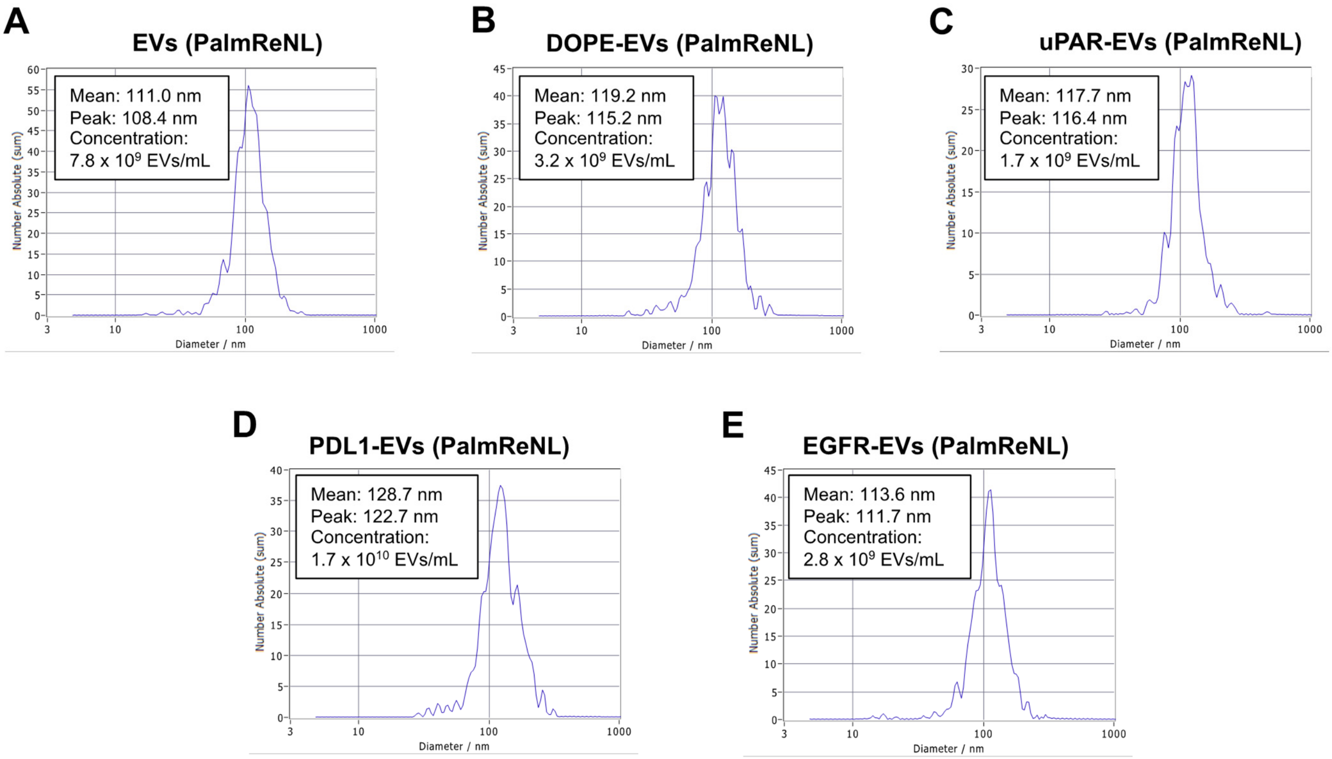


**Figure S3.** Characterization of PalmReNL-EVs engineered with THPs by nanoparticle tracking analysis (NTA). **A-B**) Unmodified and DOPE-only control PalmReNL-EVs. **C-E**) PalmReNL-EVs bearing uPAR-, PDL1-, and EGFR-binding peptides.

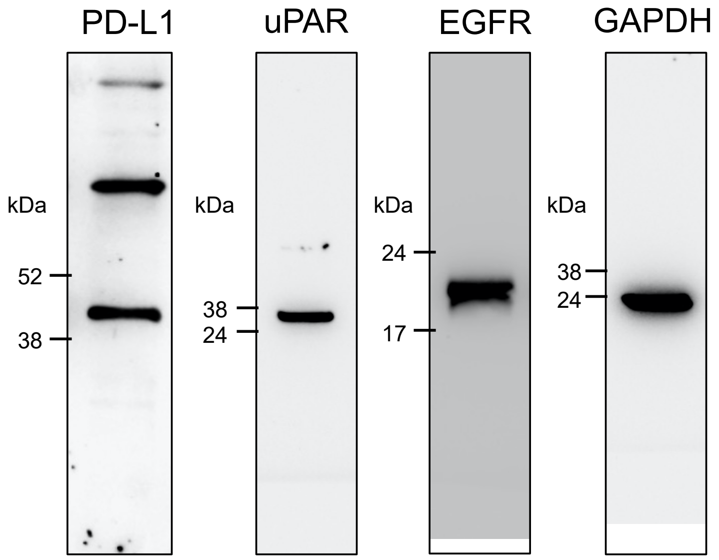


**Figure S4.** Western blot analysis of the target membrane protein expression in MDA-MB-231 cells under the non-reducing condition. GAPDH is a loading control. Original images for Figure 2D.
